# Discrete Emotions, Depressive Symptoms, and Caregiver Burden in Adult Children of Parents with Cognitive Change

**DOI:** 10.1093/geroni/igaf122.3915

**Published:** 2025-12-31

**Authors:** Dustin Gad, Jenna Wells, Joan Monin

**Affiliations:** Cornell University, Ithaca, New York, United States; Cornell University, Ithaca, New York, United States; Yale University, New Haven, Connecticut, United States

## Abstract

Family dementia caregivers often struggle with caregiver burden and higher rates of depression than similarly-aged non-caregivers. Specific negative emotions including guilt and anger have been implicated in family dementia caregivers’ mental health cross-sectionally. However, few studies have investigated discrete positive emotions and how discrete emotions influence caregivers’ mental health longitudinally. Our study addresses these gaps in N = 145 adult children of parents with cognitive change, who completed questionnaires at baseline (T1) and one year later (T2) about their emotions (PANAS), depressive symptoms (CES-D), and caregiver burden (ZBI-12). For cross-sectional associations (T1), we conducted Pearson’s correlations between each emotion and outcome. For longitudinal associations, we conducted linear regressions with each emotion at T1 predicting changes in each outcome from T1 to T2, controlling for T1 levels of the outcome. All emotions that were significantly correlated with outcome variables (p < .0025 with Bonferroni correction) were then included in a regression predicting the respective outcome variable. Cross-sectionally, greater distress, upset, shame, and fear were associated with greater depressive symptoms; greater guilt was associated with greater caregiver burden. Longitudinally, greater guilt was associated with increased depressive symptoms (B = .345); greater determination was associated with decreased caregiver burden (B = -.281). Findings suggest alleviating guilt (i.e., feeling that one has upset or harmed another person) through cognitive restructuring may help protect caregivers’ mental health; and promoting determination through caregiver skills trainings, support groups, and strength recognition techniques (e.g., benefit-finding), may mitigate caregivers’ long-term feelings of burden.

